# Effects of O_2_/Ar Ratio on Preparation and Dielectric Properties of CaZrO_3_ Films by Radio Frequency (RF) Magnetron Sputtering

**DOI:** 10.3390/ma17051120

**Published:** 2024-02-29

**Authors:** Mingjian Ding, Bing Xie, Ming Lv, Zhenya Lu

**Affiliations:** 1 School of Materials Science and Engineering, South China University of Technology, Guangzhou 510640, China; fdingfff@hotmail.com (M.D.); immlv@scut.edu.cn (M.L.); 2 College of Material Science and Engineering, Sichuan University, Chengdu 610064, China; mpxiebin@163.com

**Keywords:** CaZrO_3_ thin films, RF magnetron sputtering, annealing, dielectric properties, O_2_/Ar flow ratio, micro morphology, ohmic conduction mechanism

## Abstract

CaZrO_3_ (CZO) thin films were deposited on Pt/Ti/SiO_2_/Si substrates at 450 °C by radio-frequency magnetron sputtering technology. The microstructures and dielectric properties of CZO thin films were investigated. X-ray diffraction analysis reveals that the perovskite orthogonal CZO phase would be promoted by a higher O_2_ partial pressure in the flow ratio of O_2_/Ar after thin films were annealed at 700 °C for 3 h in air. The films prepared under the flow ratio of O_2_/Ar (20:40, 30:40 and 40:40) show the main perovskite crystal phase of CaZrO_3_ with a small amount of Ca_0_._2_Zr_0_._8_O_1_._8_. The main crystal phase was Ca_0_._2_Zr_0_._8_O_1_._8_ when the film was deposited under an O_2_/Ar ratio of 40:10. The annealed film with a 40:40 O_2_/Ar ratio exhibits a dielectric performance with a high dielectric constant (εr) of 25 at 1 MHz, a temperature coefficient of permittivity of not more than 122.7 ppm/°C from 0 °C to 125 °C, and a low leakage current density of about 2 × 10^−7^ A/cm^2^ at 30 V with an ohmic conduction mechanism.

## 1. Introduction

High dielectric constant (high-K) thin film materials have become the focus of researchers’ interest in recent years. The development of high-K dielectric thin film materials mainly serves to meet the needs in the two following areas.

(i) Development Requirements for High-Performance Chips

As the number of transistors on integrated circuits (ICs) increases according to Moore’s Law, the gate length and gate oxide thickness of complementary metal-oxide-semiconductor field transistors (CMOSFETs) decrease significantly. The International Technology Roadmap for Semiconductors (ITRS) states that transistors with feature dimensions below 100 nm require a gate oxide thickness of less than 1.5 nm [1]. A gate oxide thickness reduction to 1.5 nm at 1 V leads to leakage current exceeding 1 A/cm^2^ due to direct tunneling [2]. This indicates that problems related to gate leakage current, standby power consumption, and gate oxide reliability are exacerbated when the gate oxide thickness is drastically reduced in CMOSFETs to achieve a high performance [3]. To preserve gate capacitance and the suppress tunneling current, high-dielectric-constant materials are used to create a thick gate dielectric layer.

The gate thickness of new materials is typically defined by the equivalent oxide thickness (EOT), as shown in Equation (1).

(1) $$t_{\mathrm{OX}}=\mathrm{EOT}=\left(\frac{3.9}{K}\right)t_{\mathrm{high}-K}$$

where 3.9 is the permittivity of silicon dioxide, and K is the permittivity of the new material. It is important to note that a larger K value results in a larger t_high-K_. With the development of semiconductor technology, there is a demand for device miniaturization and high performance, which requires using new high-K dielectric materials [2].

(ii) High-Frequency Integrated Circuit and Discrete Component Requirements

As the operating frequency of ICs tends to be higher, the traditional surface-mount (SMT) components are no longer suitable due to excessive parasitic inductance or impedance mismatch. SMT capacitors are typically thick and large, which can make them difficult to mount close to the chip. When they are forced to be mounted on the circuit board, a large parasitic inductance is generated, limiting the operating frequency to 100 MHz [4]. The limited performance results in the use of more decoupling capacitors, which increases the cost of the ICs. Therefore, a film-type capacitor with a small size, high capacitance density, good temperature stability, and easy integration is required to perform high-frequency circuit decoupling and filtering. Currently, advanced 2.5-dimensional (2.5D) packaging technology integrates capacitors and other integrated passive devices (IPDs) directly on the Interposer [5,6]. This method utilizes the relatively small parasitic inductance of the integrated film capacitors to eliminate power supply noise and significantly improve signal transmission speed at higher frequencies [7].

In addition, the increasing use of microwave monolithic integrated circuits (MMICs) has led to the widespread use of discrete film capacitors with metal-insulator-metal (MIM) and metal-insulator-semiconductor (MOS) structures in radio frequency (RF) ICs because film capacitors have lower insertion loss compared to ceramic capacitors. Well-known electronic component manufacturers, such as Murata, AVX, Vishay, and Skyworks, have developed silicon oxide and silicon nitride dielectric film capacitors for use in automotive LIDAR, optical communication, and power amplifiers [8,9,10].

The existing film-type silicon capacitors are made of dielectric materials with low constants (<9), which cannot meet the requirement of capacitance density. To increase the capacitance density, three-dimensional (3D) structured silicon deep trenches with a high-aspect ratio are often used to increase the effective area of the capacitor electrodes [11,12,13,14]. However, the fabrication process of 3D structures is complex and expensive. As a result, research has focused on high-K dielectric films for planar capacitors.

Calcium zirconia (CaZrO_3_) is a perovskite-type oxide with a high melting point, low coefficient of thermal expansion, and high strength [15]. It also exhibits both orthorhombic and cubic phases. CaZrO_3_(CZO) also has excellent dielectric properties, with a dielectric constant of about 30, a low dissipation factor (tanδ) and a high thermal stability [16]. CaZrO_3_-based materials have received much attention due to their mechanical, thermal, and electrical properties [17,18,19]. The main advantages of CaZrO_3_-based materials are their high chemical and thermal stability. As a paraelectric dielectric, perovskite CZO films and ceramics are promising candidates for multilayer ceramic capacitors (MLCCs) and dielectric resonators, especially for microwave and gate applications because of their low dielectric loss, low leakage current density, low-temperature coefficient, high resistivity, high dielectric constant and high breakdown voltage [20,21]. Compared with perovskite CZO ceramics, limited work on the fabrication of CZO films has been reported, especially the study of preparation perovskite CZO thin films by radio-frequency magnetron sputtering technology.

The preparation of perovskite CZO thin films has been investigated by pulsed laser deposition (PLD) technique [1,22,23] and sol-gel technique [24,25,26]. The thermal stability and dielectric properties of CZO thin films were studied [1]. Low leakage current density and high breakdown strength were achieved in these researches. In any case, the reported dielectric constant (ε_r_) of CZO thin films (12~18) is still less than its bulk form, which may result from the remaining amorphous phase or calcia-stabilized zirconia (CSZ) phase in the films. Yaginuma et al. [23] investigated CZO dielectric films by PLD and found that the breakdown field strength of CZO dielectric films was much lower than the theoretical value, but phase structure analysis and microscopic morphology were not mentioned. Joseph M [22] also deposited amorphous thin films on a Si substrate by PLD. When the films were prepared at an oxygen partial pressure of 150 mTorr and annealed in air at 950 °C/6 h, the CZO phase was obtained, but the CSZ phase was still present. CaZrO_x_ thin films prepared by PLD were studied by H.W.LIU [1]. XRD showed that the deposited films remained amorphous after rapid thermal annealing at 700 °C. The dielectric constant of the CaZrO_x_ film was found to be about 10.5 at 1 MHz. Yu et al. [24,25,26,27] studied CZO thin films by the sol-gel method, and the dielectric constant of the CZO thin film was only 18. Based on our knowledge, the main difficulty in the preparation of perovskite CZO films is how to reduce or avoid the CSZ in the thin films. CSZ is a kind of solid electrolyte. For dielectric applications, it is important to avoid CSZ in the preparation of perovskite CZO thin films.

RF magnetron sputtering method is one of the important techniques for preparing thin films and is widely used in the preparation of advanced functional thin films. In particular, it can be flexibly used to prepare various high-quality oxide thin films with a large area. 

In this work, the radio-frequency magnetron sputtering process was employed to prepare a high-K perovskite CZO film. The influences of processing parameters, especially the oxygen content in the flow ratio, on the phase characteristics of CZO films and dielectric properties were investigated experimentally.

## 2. Materials and Methods

### 2.1. Sputtering Target Preparation

**Figure 1:**
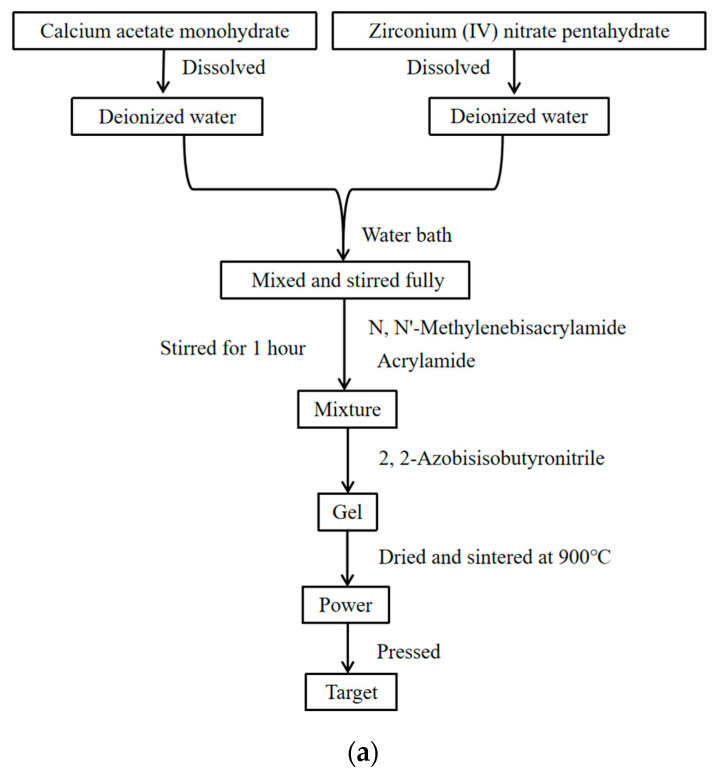

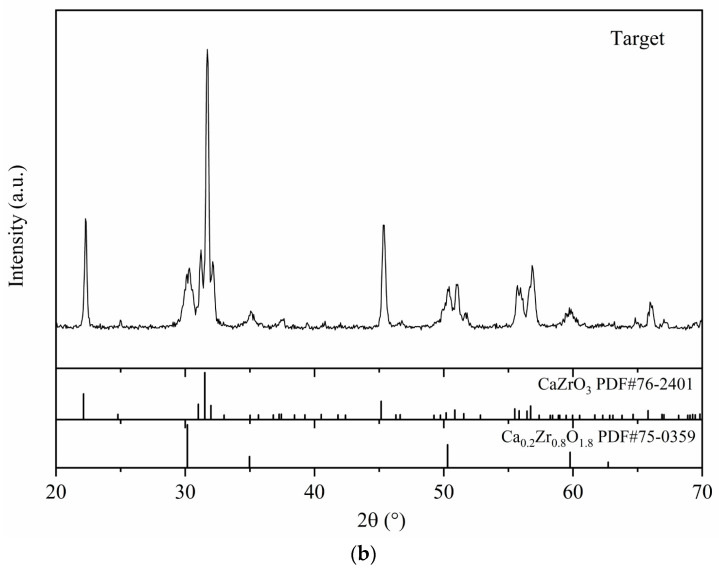
CaZr_1_._05_O_3_ target was prepared by using sol-gel method (**a**); and X-ray diffraction pattern of CaZr_1_._05_O_3_ target (**b**).

The radio-frequency (RF) magnetron sputtering process was employed to grow CZO films on Si substrates with a layer of platinum as the bottom electrode. For this work, the sputtering target was stoichiometric CaZr_1_._05_O_3_ powder synthesized using the acrylamide sol-gel method (see Figure 1a). 

High-purity, commercially available powders of Calcium acetate monohydrate (98.0% pure, Chron chemicals, Chengdu, China) and Zirconium (IV) nitrate pentahydrate (99.48% pure, Chron chemicals, Chengdu, China) were used as starting materials. These two raw materials were accurately weighed in molar ratios and fully dissolved in deionized water, respectively. The above two solutions were mixed and stirred fully using the thermostat temperature water bath with a magnetic stirrer (DF-101S, Yuhua instruments, Gongyi, China). Then, N,N′-Methylenebisacrylamide (98.5% pure, Chron chemicals, Chengdu, China) and Acrylamide (99.0% pure, Chron chemicals, Chengdu, China) were added to the mixture and stirred for 1 h. After that, we added 2,2-Azobisisobutyronitrile (99.0% pure, Chron chemicals, Chengdu, China) into it and increased the temperature to 80 °C to make the mixture concretionary. After the gel was dried, it was mixed with urea (≥99.0% pure, Chron chemicals, Chengdu, China) and ground uniformly, and sintered at 900 °C for three hours. The as-prepared powder was ground thoroughly, followed by pressing it into a cylindrical shape (60 mm in diameter × 3.5 mm in thickness) to obtain the CaZr_1_._05_O_3_ target for sputtering. X-ray diffraction results show that the composition of the CaZr_1_._05_O_3_ target is mainly CaZrO_3_ and Ca_0_._2_Zr_0_._8_O_1_._8_(CSZ) (see Figure 1b).

### 2.2. Thin Film Preparation

The base vacuity of the deposition chamber was 4.0 × 10^−4^ Pa, and the substrate was kept at 450 °C during the sputtering of the film. In order to finish the electric properties measurement, the Pt/Ti/SiO_2_/Si substrates were used in the present work. The sputtering pressure was fixed at 2.0 Pa, and the flow ratio of O_2_:Ar varied from 10:40 to 40:40. The as-deposited films were cooled down to room temperature at the base pressure before they were taken out of the deposition chamber and then annealed for 3 h at 700 °C in air (with an oxygen gas content of about 21%). The deposition parameters are shown in Table 1. 

### 2.3. Characterization of Thin Film Structure and Dielectric Properties

The phase compositions and crystallographic orientations of the as-deposited and annealed films were examined using X-ray θ-2θ scans, using X-ray diffraction (XRD, DX-2700, Dandong, China). The micro-morphology of the films was analyzed by using a scanning electron microscope (JEOL, JSM-7500F, Tokyo, Japan). Before electrical measurements, films were sputter-coated with circular Au top electrodes (Φ = 0.4435 mm in diameter) via a shadow mask in Ar at room temperature. The frequency-dependent dielectric properties (dielectric constant and loss tangent) were measured using a precision impedance analyzer (4294A, Agilent, Santa Clara, CA, USA). The current density-electric field (J-E) curves and polarization-electric field (P-E) hysteresis loops of the CZO films were measured using a Precision Premier II ferroelectric tester (Radiant Technologies, Albuquerque, NM, USA).

## 3. Results and Discussion

**Figure 2:**
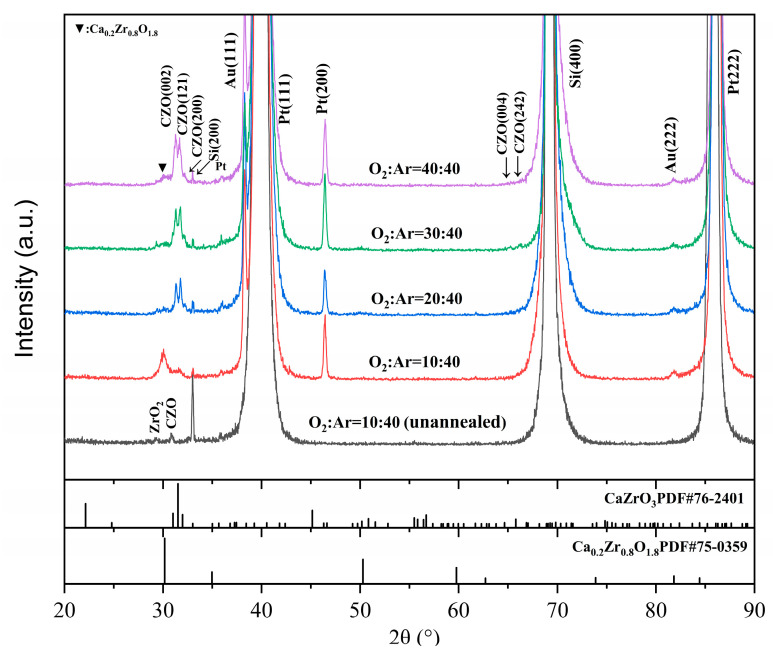
X-ray diffraction patterns of CaZrO_3_ thin films with different O_2_/Ar ratios.

Figure 2 shows the XRD patterns of the thin films prepared on Pt/Ti/SiO_2_/Si substrates with different O_2_:Ar ratios, respectively. Perovskite CZO phase is identified in all thin films, which is a perovskite orthorhombic crystal structure (reference to ICSD-PDF#76-2401). At a low O_2_/Ar ratio of 10:40, the as-deposited films were mainly CZO and ZrO_2_ with relatively weak peak intensities, but after annealing treatment, the main crystalline phase of the films became calcium-stabilized zirconia Ca_0_._2_Zr_0_._8_O_1_._8_ (CSZ) phase with a cubic crystal structure (ICSD-PDF#75-0359). Meanwhile, the peak intensity of the CZO phase also increased. This indicates that annealing can promote the reaction between the internal components of the as-deposited films and improve the crystallinity of the films in air, which is beneficial for the preparation of the films [28,29,30]. Therefore, all the films were annealed and characterized.

Under the same annealing conditions, the crystallinity and the content of the perovskite phase CZO within the annealed films are significantly improved with an increasing O_2_/Ar ratio compared to the films deposited at an O_2_/Ar ratio of 10:40. The annealed films prepared with the O_2_/Ar ratio of 20:40, 30:40, and 40:40, respectively, show the main orthorhombic crystal phase of perovskite CZO with a small amount of CSZ phase. Also, the intensities of the (002), (121) and (200) peaks of the CZO phase become gradually stronger as the oxygen content increases. This indicates that high O_2_/Ar ratios are beneficial for the formation of CZO films after annealing.

From the results in Figure 2, it is obvious that the oxygen partial pressure of the flow plays an important role in the crystallinity and the formation of CSZ and CZO phases of the annealed films. Considering that CSZ phase could be formed at 400 °C, lower than for CZO [12,31], and that sufficient oxygen atmosphere was useful to the formation of CZO phase [9,24], this may be the reason why the CSZ in the film increases and then decreases after annealing. An increase in the oxidation reaction of the species emitted from the target is useful to promote the formation of CZO and improve the crystallinity of the films after annealing; this suggests that the O_2_/Ar ratio first affects the film deposition process, which in turn promotes the reduction of the CSZ phase and the formation of the CZO phase in the films after annealing. 

**Figure 3:**
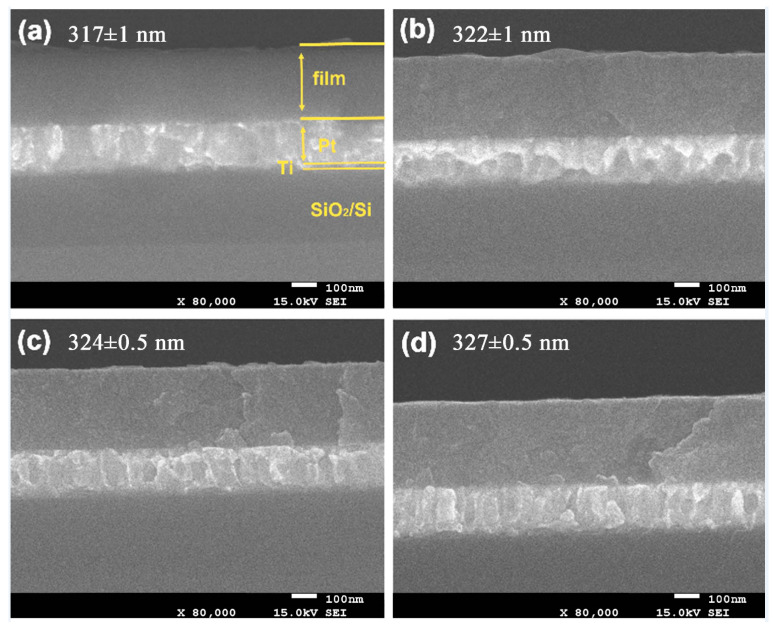
Cross-section image of the annealed films deposited on Pt/Ti/SiO_2_/Si substrates with different O_2_/Ar ratios of (**a**) 10:40, (**b**) 20:40, (**c**) 30:40 and (**d**) 40:40.

Figure 3 shows the SEM cross-section graphs of the films deposited on Pt/Ti/SiO_2_/Si substrates and annealed at 700 °C for 3 h in air (with an oxygen gas content of about 21%). One can note that the thickness of the annealed films increases with the increase of the O_2_/Ar ratio. This may be because during the CZO deposition process, the target is bombarded with species that have a high activity and react more readily with oxygen at high partial pressures of oxygen. As a result, the film contains more CaO^+^ ions, which leads to an increase in film thickness after annealing [22]. All the thicknesses of the thin films are more than 310 nm, and the thin films exhibit a similar morphology in the cross-section and interface. It is agreed that all the annealed thin films are dense and crack-free. Furthermore, the thicknesses of the films are a little bit different, even though all the thin films were deposited at the same time. In addition, the surface of the films has some undulations, and the flatness of the films’ surface is best at O_2_/Ar = 40:40. When combining the results of the XRD measurement (Figure 2), the difference in the thickness and microscopic morphology of thin films should be attributed to the difference in microstructure of the annealed films, which results from different O_2_/Ar ratios during the preparation of these thin films.

**Figure 4:**
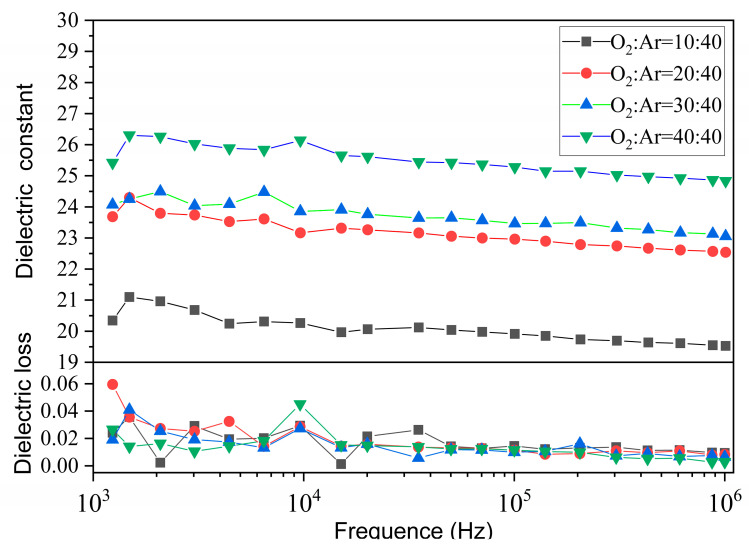
Dielectric constant and dielectric loss dependence on frequency for the CaZrO_3_ films annealed at 700 °C in air.

All the as-deposited thin films were annealed at 700 °C in air atmosphere, and their dielectric properties as a function of the frequency was measured at room temperature, respectively. All thin films show a weak dielectric constant (ε_r_) dependence on frequency at room temperature (see Figure 4). Besides this, the annealed film prepared under 40:40 O_2_/Ar ratios exhibits the highest dielectric constant (ε_r_~26), while the annealed film prepared under 10:40 O_2_/Ar ratios shows the lowest dielectric constant (~20), ranging from 1 kHz to 1 MHz, which is larger than for the films prepared by PLD and Sol-Gel [1,27]. Moreover, the dielectric constant of the films tends to become larger with the increase of the O_2_/Ar ratio after annealing. The dielectric loss of all films is also very small, essentially below 0.004 over the tested frequency range. The characterization results of the dielectric properties of the annealed films further demonstrate the importance of the oxygen content during the deposition of thin films.

**Figure 5:**
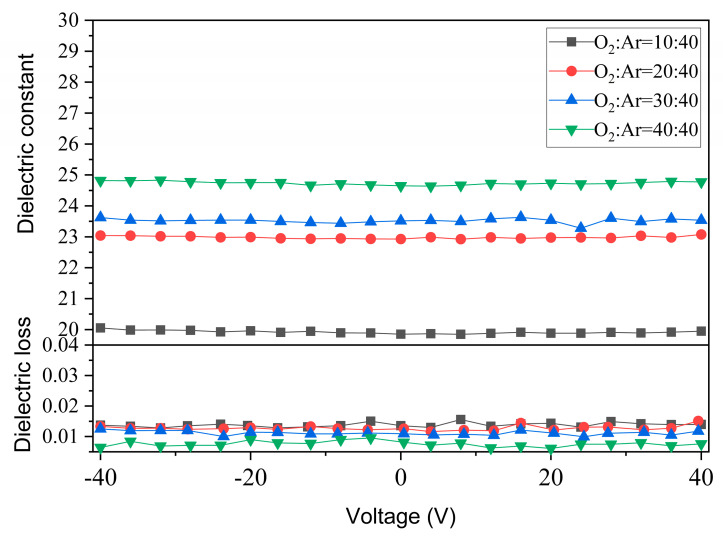
Dielectric constant and dielectric loss dependence on DC bias voltage for the CaZrO_3_ films annealed at 700 °C in air.

Figure 5 shows the bias characteristics of the annealed films at DC voltage. The results of the dielectric constant versus applied DC voltage of thin films at 100 kHz are shown in Figure 5. All the films show a typical paraelectric characteristic and could withstand 40 V DC voltage steadily, and the electric field strength reached about 1.25 MV/cm. This metal-insulating dielectric film-metal structure (MIM) capacitor has a good voltage stability over the bias voltage range of −40 V to +40 V, and the rate of change of the dielectric constant is only 0.77% at the O_2_/Ar ratio of 40:40, indicating that CZO is a linear dielectric material. Moreover, the dielectric loss of all annealed films is also less affected by the applied voltage, which is basically below 0.015. Among them, the sample with an O_2_/Ar ratio of 40:40 is the best, at less than 0.01.

**Figure 6:**
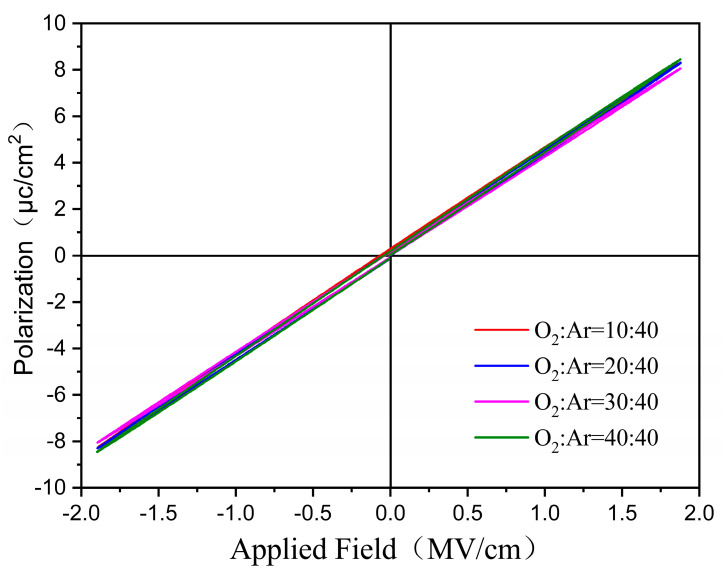
P–E hysteresis loops of the annealed films prepared with different O_2_/Ar ratios.

The polarization loops of all annealed films were measured under an electric field using a Radiant Precision Workstation (Radiant Technologies, Albuquerque, NM, USA), and they are shown in Figure 6. The results reveal a clear linear function between the polarization and electric field, which is the typical paraelectric characteristics and coincides with the results shown in Figure 5. This also indicates that all thin films could withstand an AC field strength of about 1.8 MV/cm.

**Figure 7:**
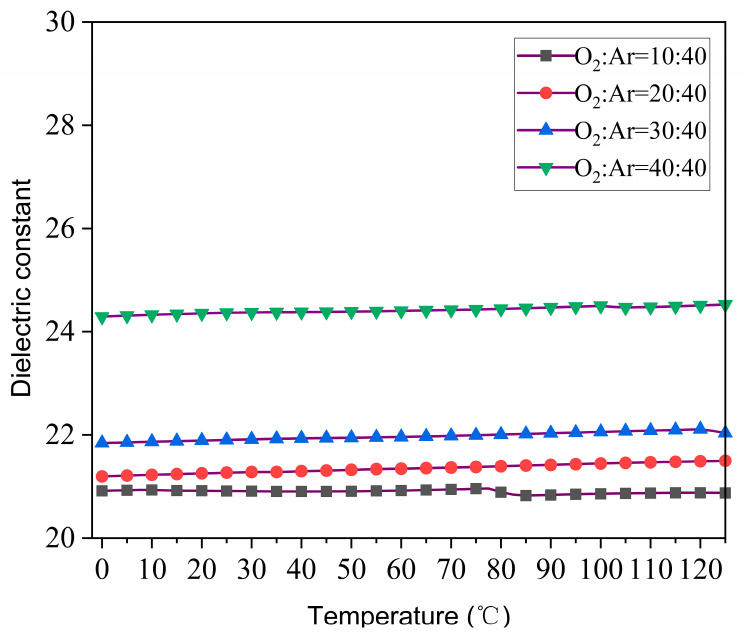
Dielectric constant dependence on temperature for the CaZrO_3_ films annealed at 700 °C in air.

The temperature dependence of the dielectric constant in the range of 0~125 °C is shown in Figure 7. All the annealed films exhibit typical linear dielectric characteristics in the range of 0~125 °C. Also, the thin film prepared under a 40:40 O_2_/Ar ratio shows the highest dielectric constant among the annealed films in the range of the measured temperature. For capacitors, the temperature coefficient of capacitance (TCC) is an important product technical indicator. The temperature sensitivity of the materials used for capacitors determines the environmental conditions under which the capacitors can be used. 

The temperature coefficient of permittivity (Tε) can be calculated by the following formula:

(2) $$T\varepsilon =\frac{\varepsilon_{2}-\varepsilon_{1}}{\varepsilon_{1}\left(T_{2}-T_{1}\right)}$$

In Equation (2), ε_1_ represents the measured dielectric constant at T_1_, and ε_2_ represents the measured dielectric constant at T_2_; normally, T_1_ is taken at 25 °C. It is interesting to note that the capacitance varies very little over the temperature range of 0 °C to +125 °C (see Table 2), which demonstrates the excellent temperature stability of CSZ and CZO films.

**Figure 8:**
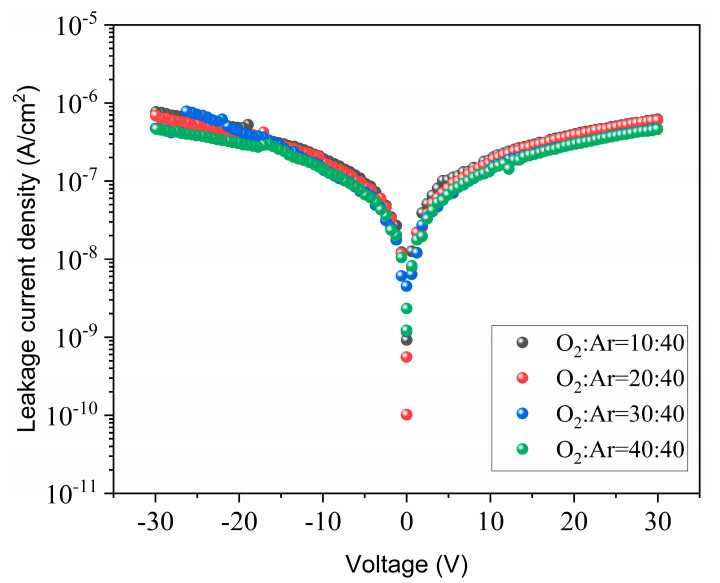
Leakage current densities of annealed CaZrO_3_ films prepared with different O_2_/Ar ratios.

Figure 8 illustrates the leakage current properties of the annealed films. It shows that all the leakage current densities of the thin films are less than 1 × 10^−6^ A/cm^2^ and that the leakage current densities of thin films deposited under 30:40 and 40:40 O_2_/Ar ratios are a little bit lower than for the other two thin films. Moreover, the leakage current has a good symmetry under positive and negative bias. The annealed film prepared at a 40:40 O_2_/Ar ratio shows the lowest leakage current density, which is about 2.0 × 10^−7^ A/cm^2^ at 30 V. This may be attributed to the fact that as the O_2_/Ar rate increases, the as-deposited film is more fully oxidized during the deposition process, leaving fewer oxygen vacancies inside the film after annealing, which in turn reduces the leakage current.

To further understand the leakage mechanism of the annealed film prepared at a 40:40 O_2_/Ar ratio, we have fitted the current-voltage curve measured at room temperature. According to Ohm’s law [32]:

(3) J=σE

where J is the leakage current density, σ is the conductivity, and E is the electric field strength. Take the logarithm on both sides to obtain:

(4) lgJ=lgE+lgσ

**Figure 9:**
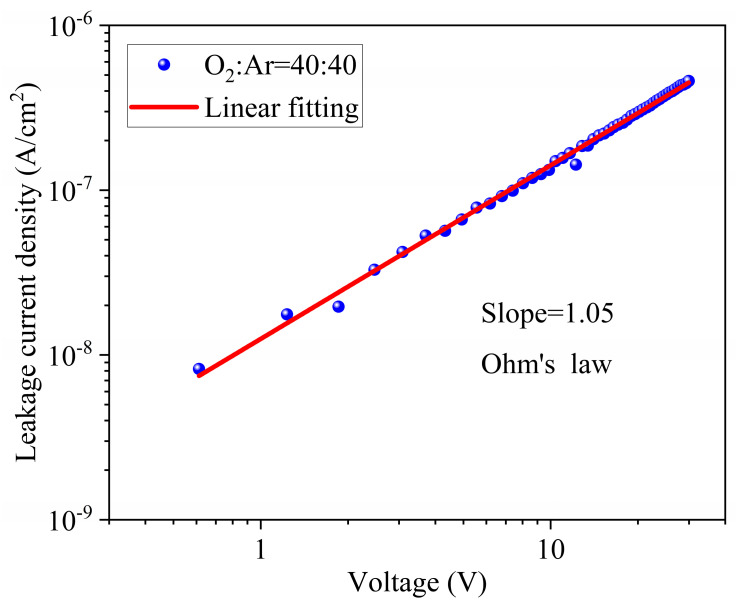
The current-voltage fitting curve of the thin film prepared at a 40:40 O_2_/Ar ratio.

According to the fitting results in Figure 9, it can be seen that lgJ and lgE have a linear relationship from an applied voltage range of 0 to 30 V, and the slope of the fitting curve is 1.05, which is consistent with the ohmic conduction mechanism.

The second phase, CSZ, is a film containing oxygen vacancies formed by calcium replacing zirconium in zirconia, which improves the quality of the film by reducing its content in the annealed film [33]. The result of XRD suggests that a good crystallinity and the highest perovskite phase content should be responsible for the best dielectric properties of the annealed film deposited at a 40:40 O_2_/Ar ratio. One can agree that the thin film deposited at a 40:40 O_2_/Ar ratio shows the best dielectric properties among all of the annealed films in the present research. It exhibits the highest dielectric constant, a good temperature stability and the lowest leakage current density. 

## 4. Conclusions

Perovskite CaZrO_3_ thin films were successfully fabricated on Pt/Ti/SiO_2_/Si substrates at 450 °C by radio-frequency magnetron sputtering technology. It is demonstrated that the O_2_/Ar ratio plays an important role in the formation of the perovskite CZO phase in the films after annealing at 700 °C for 3 h in air. Ca_0_._2_Zr_0_._8_O_1_._8_ phase could be suppressed effectively by a high O_2_/Ar ratio, and the perovskite CZO thin films could be obtained by a 40:40 O_2_/Ar ratio. The perovskite microstructure leads to an annealed CZO thin film with ε_r_ ~25, tanδ ~0.003 at 1 MHz, Tε not greater than 122.7 ppm/°C from 0 °C to 125 °C, and a leakage current density ~2 × 10^−7^ A/cm^2^ at 30 V with the ohmic conduction mechanism.

## Figures and Tables

**Table 1:** Deposition parameters of CZO films prepared by radio-frequency magnetron sputtering process.

Parameters	
Base pressure (Pa)	4.0 × 10^−4^
Substrate temperature (°C)	450
Sputtering pressure (Pa)	2.0
Sputtering power (W)	70
Deposition time (min)	300
Post-deposition cooling condition	4.0 × 10^−4^ Pa, naturally
Flow ratio of O_2_:Ar	10:40, 20:40, 30:40, 40:40
Annealing condition	700 °C × 3 h, 2 °C/min

**Table 2:** TCC of films prepared with different O_2_/Ar atmospheres.

Tε (Sample)	Unit	0 °C	125 °C
TCC(O_2_:Ar = 10:40)	ppm/°C	−5.58	−17.58
TCC(O_2_:Ar = 20:40)		136.7	108.1
TCC(O_2_:Ar = 30:40)		109.8	60.1
TCC(O_2_:Ar = 40:40)		122.7	65.7

## Data Availability

Data are contained within the article.
